# Cerebrolysin alleviates early brain injury after traumatic brain injury by inhibiting neuroinflammation and apoptosis via TLR signaling pathway

**DOI:** 10.1590/acb370605

**Published:** 2022-09-05

**Authors:** Weihong Lu, Zhonghua Zhu, Dongliang Shi, Xiaoyu Li, Jingzhi Luo, Xingzhi Liao

**Affiliations:** 1 BS. 904th Hospital of Joint Logistic Support Force of PLA – Department of Anesthesiology – Wuxi, China.; 2 MD. Anhui Medical University – Wuxi Clinical College – 904th Hospital of Joint Logistic Support Force of PLA – Department of Neurosurgery – Wuxi, China.

**Keywords:** Brain Injuries, Traumatic, Neuroinflammatory Diseases, Apoptosis

## Abstract

**Purpose:**

Traumatic brain injury (TBI) is a major cause of death and disability. Cerebrolysin (CBL) has been reported to be anti-inflammatory by reducing reactive oxygen species (ROS) production. However, the neuroprotection of CBL in TBI and the potential mechanism are unclear. We aimed to investigate the neuroprotection and mechanisms of CBL in TBI.

**Methods:**

The TBI model was established in strict accordance with the Feeney weight-drop model of focal injury. The neurological score, brain water content, neuroinflammatory cytokine levels, and neuronal damage were evaluated. The involvement of the early brain injury modulatory pathway was also investigated.

**Results:**

Following TBI, the results showed that CBL administration increased neurological scores and decreased brain edema by alleviating blood‑brain barrier (BBB) permeability, upregulating tight junction protein (ZO‑1) levels, and decreasing the levels of the inflammatory cytokines tumor necrosis factor‑α (TNF‑α), interleukin‑1β (IL‑1β), IL‑6, and NF‑κB. The TUNEL assay showed that CBL decreased hippocampal neuronal apoptosis after TBI and decreased the protein expression levels of caspase‑3 and Bax, increasing the levels of Bcl‑2. The levels of Toll‑like receptor 2 (TLR2) and TLR4 were significantly decreased after CBL treatment. In TBI patients, CBL can also decrease TNF‑α, IL‑1β, IL‑6, and NF‑κB levels. This result indicates that CBL‑mediated inhibition of neuroinflammation and apoptosis ameliorated neuronal death after TBI. The neuroprotective capacity of CBL is partly dependent on the TLR signaling pathway.

**Conclusions:**

Taken together, the results of this study indicate that CBL can improve neurological outcomes and reduce neuronal death against neuroinflammation and apoptosis via the TLR signaling pathway in mice.

## Introduction

Traumatic brain injury (TBI) is still a major public health problem and a major cause of death and disability that imposes a substantial economic burden worldwide. TBI has a high incidence in low-income and middle-income countries^1^-^3^. The incidence of TBIs is increasing rapidly due to the significant increase in road traffic collisions, including motor vehicle accidents and fall injuries in the elderly population^3^. An increasing number of studies have found that long-term outcomes have substantially improved with the advance of medical standards, while no drug interventions can improve early brain injury after TBI^2^-^8^. Hence, studies aiming to further clarify the pathophysiological mechanisms of TBI and search for effective pharmacological intervention targets are very important and necessary. The pathophysiology of TBI includes several different physiological changes and mainly involves primary brain injury and secondary brain injury after TBI^9^. Secondary brain injury, including calcium overload, oxidative stress, neuroinflammation, and apoptosis, can be reversed^10^,^11^. According to previous studies^12^-^14^, inhibition of oxidative stress and neuroinflammation decreases mitochondrial apoptosis, improves neurological function, and decreases cerebral edema after TBI. Currently, the neuroprotective effect of the inhibition of neuroinflammation and apoptosis remains unclear.

Cerebrolysin (CBL) is a small molecule peptide extracted from the porcine brain and has been previously used as a nootropic drug^15^. Increasing studies have demonstrated that CBL administration can promote recovery of motor function, improve early brain injury (EBI), and decrease hippocampal neuronal death^16^-^18^. DeBoer *et al.* 16 reported that poststroke CBL administration leads to recovery of motor function independent of rehabilitative training without a protective effect on stroke volume in a stroke model. In a seizure model, Kang *et al.* 15 also confirmed that CBL can decrease hippocampal neuronal death after the seizure. In recent clinical studies, CBL improved overall outcomes after moderate to severe TBI patients^19^,^20^ and was also safe and better for early rehabilitation patients after ischemic stroke^21^. However, the neuroprotective effects of CBL treatment on TBI are controversial. The neuroprotective mechanisms of CBL are also unclear.

TBI develops from reactive oxygen species (ROS), an inflammatory cytokine, and glutamate-induced excitotoxicity, which can lead to neuronal death quickly in the brain^14^,^22^. The molecular signaling pathways of neuroinflammation and apoptosis are complex. Toll-like receptor (TLR), an innate immune receptor of bacterial endotoxins, then accumulates to activate the nuclear factor kappa B (NF-κB) signaling transduction pathway, which was reported to be directly involved in inflammation and apoptosis in an epilepsy model^23^, myocardial infarction model^24^, and TBI^25^. As brain tissue consumes more oxygen than most organs, more ROS are generated under oxidative stress after TBI^25^, subarachnoid hemorrhage^26^, or ischemia-reperfusion injury^27^. A further study of new potential drug targets in neuroinflammation and apoptosis by targeting the TLR signaling pathway is valuable.

In the present study, we constructed a mouse TBI model to study the effects of CBL on EBI and explored the crosstalk between neuroinflammation and apoptosis. We also explored the mechanism by which the TLR signaling pathway may regulate this process.

## Methods

The study protocol was approved by the Anhui Medical University Affiliated Wuxi Clinical College Clinical Research Ethics Committees (2021-YXLL-013). The study protocol received Ethics Committee approval from all participating centers. Written informed consent was obtained from patients whose competence was established by their accurate orientation for time, place, and person, as well as an understanding of the recruiter’s description of the trial or otherwise from their next of kin or their legal representative.

All animal experiments performed for this study complied with the National Institutes of Health guidelines for the handling of laboratory animals and were approved by the Ethics Committee of the Wuxi Medical College of Anhui Medical University (YXLL-2021-013). A total of 69 healthy adult male C57BL/6J mice (n = 23/group; age, 8-10 weeks; Anhui Medical University, Hefei, China) weighing between 22-25 g were used when conducting all the experiments for the current study. The mice were housed in animal care facilities with a 12-hour light/dark cycle and had free access to food and water.

### Animal traumatic brain injury model

The TBI model was established in strict accordance with the Feeney weight-drop model of focal injury^28^,^29^. Briefly, the mice were anesthetized with an intraperitoneal injection of 1% sodium pentobarbital (40 mg/kg) and then placed in a brain stereotaxic apparatus. The rectal temperature was maintained at 37 ± 0.5°C during the operation using a heating pad. Then, a burr hole was generated in the left hemisphere at the following coordinates: 0.2 mm posterior, 1 mm lateral, and 2.2 mm below the horizontal plane of the bregma. The bone flap was removed to expose the dura mater. The dura was placed under a weight-drop device with an impact sensor. A metal (weight 240 g, tip diameter 3 mm) was dropped from 1 cm above the dura onto the dura mater through a catheter. Then, the scalp was closed, and the mice were removed from the apparatus. Finally, the hole was covered with medical bone wax. The animals in the sham group received similar surgical procedures, but without weight-drop impact.

### Drug preparation and administration

**Figure 1:**
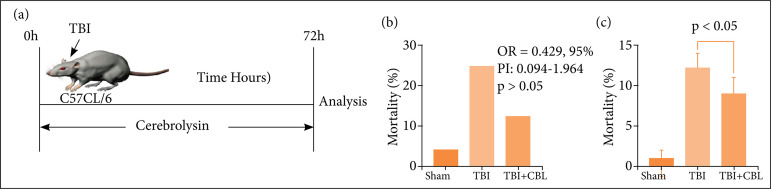
The effects of TBI on mortality and neurological function. **(a)** Schematic of the experimental paradigm for modeling TBI in mice. **(b)** The mortality rates were increased in the TBI group and decreased after CBL treatment,but there was no significant difference compared with the TBI group. **(c)** Neurological scores of mice in the shamgroup, TBI group, and TBI + CBL group at 72 h after TBI (n = 10, p < 0.05). Analysis of variance; mean ± SEM.

TBI: traumatic brain injury; CBL: cerebrospinal; SEM: standard error of mean.

After the TBI model was established successfully, animals were given daily intraperitoneal injections of either cerebrolysin (CBL, 2.5 mL/kg/day, no dilution; EBEWE Arzneimittel, Austria) or plain (control) saline for 72 hours. Figure 1 summarizes the timeline of the experimental protocol of the study.

### Neurobehavioral assessment

The severity of brain injury was evaluated by determining neurological function 72 hours after TBI using our previously described neurological grading system^30^. The neurological scores ranged from 3 to 18 and included spontaneous activities (0-3), movement symmetry of all limbs (0-3), forelimb outstretching (0-3), body proprioception (1-3), response to vibrissae touch (1-3), and climbing (1-3). All mice from every group received a behavioral assessment, and a higher score represented better neurological function.

### Brain water-content measurement

The severity of brain edema was evaluated by measuring the brain water content using the standard wet-dry method, as previously reported^30^-^32^. The mice were sacrificed 72 hours after TBI, and the entire brain was harvested and separated into the cortex, basal ganglia, and cerebellum (wet weight). Then, brain specimens from each group were dehydrated at 105°C for 24 hours to acquire the dry weight. The percentage of brain water content was equal to (wet weight – dry weight)/wet weight × 100%.

### Evans blue extravasation

Evans blue extravasation was performed as previously described^33^. Briefly, mice were anesthetized by pentobarbital sodium (50 mg/kg) injection 72 h after TBI. Evans blue dye (2%, 5 mL/kg; Sigma–Aldrich, St. Louis, MO, United States of America) was injected into the left femoral vein over 2 min and circulated for 60 min. Then, the mice were sacrificed with 100 mg/kg sodium pentobarbital via intraperitoneal injection and with phosphate-buffered saline (PBS) intracardial perfusion. The brains were removed and quickly divided into the left and right cerebral hemispheres, weighed, homogenized in saline, and centrifuged at 15,000 × g for 30 min. Subsequently, the resultant supernatant was added to an equal volume of trichloroacetic acid, incubated overnight at 4°C, and centrifuged at 15,000 × g for 30 min. Next, the resultant supernatant was collected and spectrophotometrically quantified at 610 nm for Evans blue dye.

### Analysis of reactive oxygen species

The nonfluorescent diacetylated 2’,7’-dichlorofluorescein (DCF-DA) probe (Sigma–Aldrich), which becomes highly fluorescent upon oxidation, was used to evaluate intracellular ROS production according to the manufacturer’s instructions^34^.

### Cytokine measurements of ipsilateral cortex tissue

The levels of interleukin-1β (IL-1β) (cat. no. ab197742; Abcam), IL-6 (cat. no. ab222503; Abcam), tumor necrosis factor-α (TNF-α) (cat. no. ab208348; Abcam), and NF-κB (cat. no. ab176663; Abcam) were measured by enzyme-linked immunoassay (ELISA) according to the manufacturer’s instructions^31^.

### TUNEL staining

A TUNEL assay was conducted to assess neuronal death in the brain cortex according to previously described methods^30^. TUNEL reaction mixture (50 μL) was added to each sample, and the slides were incubated in a humidified dark chamber for 60 min at 37°C. The slides were then incubated with DAPI for 5 min at room temperature in the dark to stain the nuclei, followed by imaging with a fluorescence microscope. The procedure was performed according to the manufacturer’s instructions with a TUNEL staining kit. A negative control (without the TUNEL reaction mixture) was used. The cell count was confirmed in four randomly selected high-power fields, and the data obtained from each field were averaged.

### Western blot analysis

Western blot analyses were performed as previously described^32^. Briefly, cerebral tissue samples were collected, homogenized, and separated by sodium dodecyl sulfate–polyacrylamide gel electrophoresis on 10% polyacrylamide gels. A BCA Protein Assay Kit (Beyotime) was used to measure protein concentrations with the bicinchoninic acid method. After separation, protein samples were transferred onto Immobilon nitrocellulose membranes. The membranes were blocked with 5% nonfat milk at room temperature for 1 h. The membranes were then incubated with the following primary antibodies overnight at 4°C: rabbit anti-β-actin (1:1,000, rabbit polyclonal, Abcam, ab8227), rabbit anti-Cleaved-Caspase-3 (1:5,000, Abcam, ab214430), rabbit anti-TLR2 (1:1,000, ab213676), and rabbit anti-TLR4 (1:1,000, rabbit polyclonal, Abcam, ab13556). After washing the membranes with Tris buffered saline tween (TBST) three times, horse radish peroxidase (HRP)-conjugated goat anti-rabbit IgG or goat anti-mouse IgG secondary antibodies (1:5,000) were applied, and the membranes were incubated with the secondary antibodies at room temperature for 1.5 h. The protein bands were detected using a Bio-Rad imaging system (Bio-Rad, Hercules, CA, United States of America) and quantified with ImageJ software.

### Statistical analysis

All experiments were repeated more than three times, and the data are expressed as the means and stardard error of mean (SEM). Statistical Package for the Social Sciences (SPSS) 14.0 (SPSS, Chicago, IL, United States of America) and GraphPad Prism 6 (GraphPad Software, San Diego, CA, United States of America) were used for the statistical analyses. Student’s t-test was used if two groups were compared, and a one-way analysis of variance (ANOVA) followed by Bonferroni’s post-hoc test was used if two independent variables were compared. For non-normally distributed data and/or non-homogeneous variance, we used the Kruskal–Wallis test followed by Dunn’s post-hoc test. For all statistical analyses, data were considered significant at p < 0.05.

## Results

### The effects of traumatic brain injury on mortality and neurological function

We constructed the TBI model and CBL treatment after TBI (Fig. 1a). We evaluated the effect of CBL treatment on long-term neurological damage parameters, including mortality rates and neurological scores. As shown in Fig. 1, mortality rates (Fig. 1b) were decreased in the TBI + CBL group, but there was no significant difference compared with the TBI group (odds ratio – OR = 0.429, 95% confidence interval – 95%CI – 0.094-1.964, p > 0.05). Neurological scores decreased significantly after TBI, and CBL treatment significantly increased neurological scores (p < 0.05) (Fig. 1c).

### Cerebrolysin alleviates brain edema and blood-brain barrier permeability after traumatic brain injury

**Figure 2:**
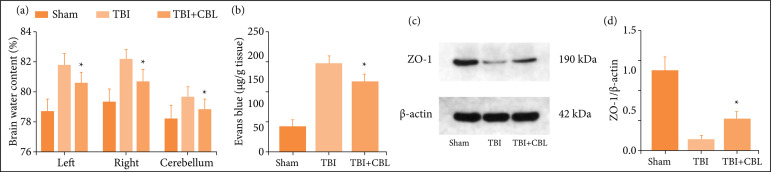
CBL alleviates brain edema and BBB permeability after TBI. **(a)** CBL alleviates brain water content after TBI. **(b)** CBL alleviates BBB permeability after TBI. **(c)** Expression of ZO-1 in the brain cortex of mice after TBI wasdetermined by Western blotting. **(d)** Quantification of ZO-1 in the brain cortex compared to the β-actinloading control. CBL increased ZO-1 expression after TBI in mice (n = 6; ANOVA; mean ± SEM).

TBI: traumatic brain injury; CBL: cerebrolysin; BBB: blood-brain barrier; ANOVA: analysis of variance; SEM: standard error of mean; *p < 0.05 *vs*. TBI group.

We used brain water content by the wet-dry method at 72 h after TBI to evaluate brain damage. The results showed that TBI significantly increased the brain water content, which was alleviated after CBL treatment (Fig. 2a). Similar results were obtained for blood-brain barrier (BBB) permeability, which was increased significantly after TBI, and CBL administration was significantly alleviated (Fig. 2b). To further clarify the BBB integrity, we detected the expression levels of Zonula occludens-1 (ZO-1) by Western blotting (Fig. 2c). The results showed that the expression of ZO-1 significantly decreased after TBI (p < 0.05 *vs*. the control group) and increased after CBL treatment (Fig. 2d). This result suggests that TBI-induced BBB permeability was disrupted, and CBL treatment markedly alleviated this effect at 72 h.

### Cerebrolysin alleviates neuronal apoptosis after traumatic brain injury

**Figure 3:**
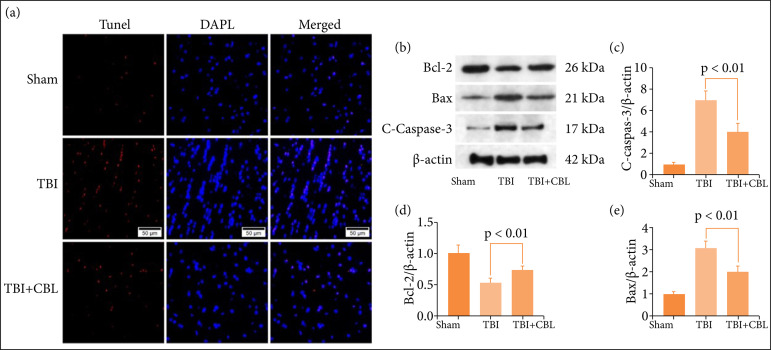
CBL alleviates brain cortical neuronal apoptosis after TBI. **(a)** TUNEL staining showed that CBL alleviated neuronal death in the brain cortex 72 h after TBI. **(b)** The expression of cleaved caspase-3, Bax, and Bcl-2 in thebrain cortex of mice after TBI was determined by Western blotting. Quantification of **(c)** cleaved caspase-3,**(d)** Bcl-2, and **(e)** Bax in the brain cortex in response to the β-actin loading control. CBL decreasedcleaved caspase-3 and Bax expression in the brain cortex and significantly increased theexpression level of Bcl-2 after TBI. p < 0.01. Scale bar = 50 μm. ANOVA; mean ± SEM.

CBL: cerebrolysin; TBI: traumatic brain injury; BBB: blood-brain barrier; ANOVA: analysis of variance; SEM: standard error of mean;DAPI: 4',6-diamidino-2-phenylindole; TUNEL: terminal deoxynucleotidyl transferase dUTP nick end labeling.

The TUNEL assay results revealed more hippocampal neuronal death after TBI, and CBL decreased neuronal apoptosis (Fig. 3a). The apoptosis-related proteins caspase-3, Bax, and Bcl-2 were detected by Western blotting (Fig. 3b), and the results showed the expression levels of caspase-3 (Fig. 3c) and Bax (Fig. 3d) decreased significantly after CBL treatment. The expression level of Bcl-2 (Fig. 3e) increased significantly after CBL treatment. Based on these results, CBL exerts neuroprotective effects after TBI.

### Cerebrolysin alleviates neuroinflammation after traumatic brain injury

**Figure 4:**
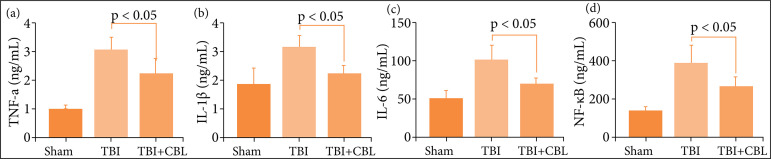
CBL alleviates neuroinflammation after TBI. CBL significantly reduced hippocampal **(a)** TNF-α, **(b)** IL-1β, **(c)** IL-6, and **(d)** NF-κB levels at 72 h after TBI (n = 6, P < 0.05, analysis of variance; means ± standard error of mean).

CBL: cerebrolysin; TBI: traumatic brain injury; TNF-α: tumor necrosis factor-α; IL: interleukin.

As previous studies have identified a vital role for neuroinflammation in EBI after TBI, increased neuroinflammation aggravates EBI^10^,^35^-^37^. The levels of proinflammatory cytokines were increased significantly after TBI, while the levels of proinflammatory cytokines decreased significantly after CBL treatment (Fig. 4a-d). Hence, these results suggested that CBL exhibited potent anti-inflammatory activity against TBI-induced neuroinflammation.

### Cerebrolysin regulates neuroinflammation and apoptosis by modulating the toll-like receptor signaling pathway after traumatic brain injury

**Figure 5:**
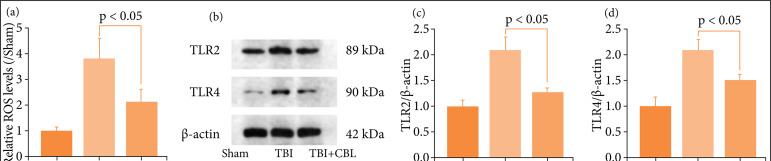
CBL regulates neuroinflammation and apoptosis by modulating the TLR signaling pathway after TBI. **(a)** The levels of ROS by the DCF-DA method. **(b)** The expression of TLR2 and TLR4 in the brain cortex of mice after TBI was determined by Western blotting. (c-d) Quantification of TLR2 and TLR4 in the brain cortex compared to the β-actin loading control. CBL significantly decreased brain cortex TLR2 and TLR4 expression after TBI (n = 6, p < 0.05; analysis of variance; mean ± SEM).

CBL: cerebrolysin; TLR: toll-like receptor; TBI: traumatic brain injury; ROS: reactive oxygen species;DCF-DA: diacetylated 2’,7’-dichlorofluorescein; SEM: stardard error of mean.

TLR is a core signaling pathway of neuroinflammation and apoptosis^38^,^39^. We explored whether the neuroprotection of CBL regulates neuroinflammation and apoptosis by modulating the TLR signaling pathway. First, we detected the ROS levels by DCF-DA probe (Fig. 5a), and the levels of ROS were increased after TBI, but decreased significantly after CBL treatment. We detected the levels of the TLR2 and TLR4 proteins by performing Western blotting (Fig. 5b). The levels of TLR2 and TLR4 were increased significantly in the TBI group and decreased after CBL administration (Figs. 5c-d). Thus, these results showed that CBL may exert neuroprotective effects by regulating the TLR signaling pathway.

### Cerebrolysin decreases the levels of proinflammatory cytokines in traumatic brain injury patients

**Figure 6:**
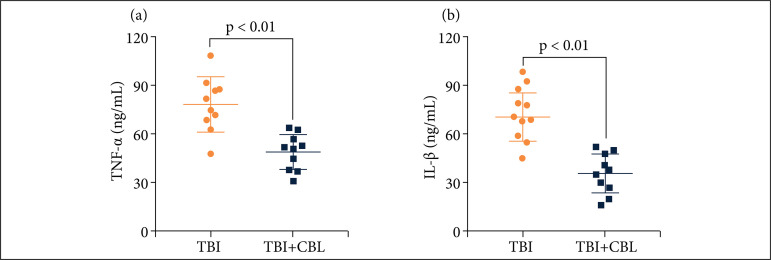
CBL alleviates neuroinflammation in TBI patients. CBL significantly reduced TBI patients’ serum **(a)**TNF-α, **(b)** interleukin-1β (IL-1β) levels at 72 h after TBI (n = 10, P < 0.01, analysis of variance; means ± SDs).

CBL: cerebrolysin; TBI: traumatic brain injury; TNF-α: tumor necrosis factor-α; IL: interleukin; SD: standard deviation.

To verify the anti-neuroinflammatory effect of CBL in TBI patients, we evaluated the expression levels of serum IL-1β, IL-6, TNF-α, and NF-κB in TBI patients. We measured the serum levels of IL-1β, IL-6, TNF-α, and NF-κB using ELISAs. The levels of proinflammatory cytokines were increased significantly after TBI, while the levels of proinflammatory cytokines decreased significantly after CBL treatment (Figs. 6a-b).

## Discussion

**Figure 7:**
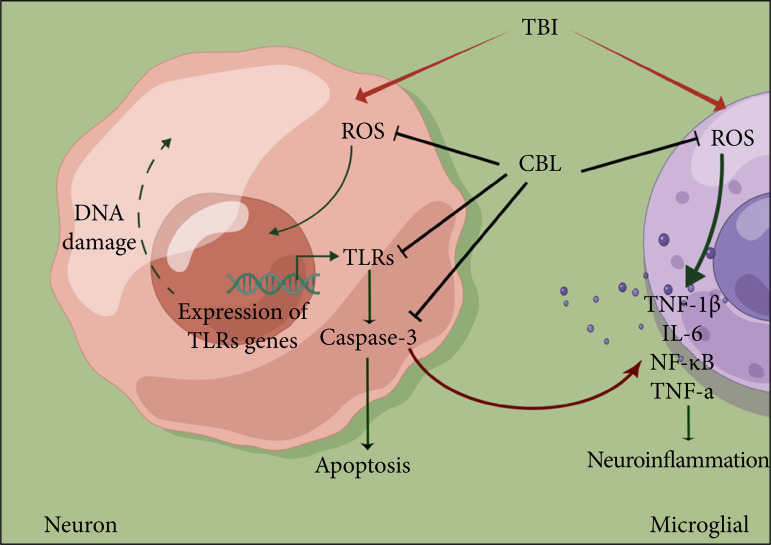
A diagram of the proposed model explaining the observations of TLR-mediatedregulation of neuroinflammation and apoptosis after TBI and the potentialmechanisms underlying the effect of CBL intervention.

ROS: reactive oxygen species; CBL: cerebrolysin; TLR: toll-like receptor; TNF: tumor necrosis factor; IL: interleukin.

Here, we evaluated the therapeutic potential of CBL to alleviate early brain injury after TBI. As shown in the present study, we found that CBL can improve neurological dysfunction and brain damage after TBI, relieve neuroinflammation and prevent apoptosis after TBI, and the antiapoptotic and antineuroinflammatory effects of CBL may be related to the TLR signaling pathway (Fig. 7).

CBL is an intravenously administered small molecule peptide extracted from the porcine brain that is composed of approximately 15% low molecular weight peptides and 85% amino acids in an aqueous solution and has been used as a nootropic drug^40^. It is a brain-specific pleiotropic agent that is proposed to target multiple pathways to improve functional recovery after neurological diseases^15^-^19^. Satou *et al.* 41 reported that CBL can promote neurite outgrowth and cholinergic fiber regeneration *in vitro*. Guan *et al.* 42 also demonstrated that CBL can alleviate brain injury after focal cerebral ischemia by regulating neuroinflammation, partly via the activation of the CREB/PGC-1α pathway. In spontaneously hypertensive rats with hyperglycemia, CBL can also alleviate the reduction in the number of dendritic spines in the prefrontal cortex and hippocampus^43^.

In clinical studies, Muresanu *et al.* 19 reported that CBL treatment is effective and safe for moderate to severe traumatic brain injury patients in a phase IIIb/IV single-center study that enrolled 142 patients. An observational retrospective clinical study also confirmed that CBL may improve the level of consciousness in stroke patients in a minimally conscious state^44^. A recent randomized, placebo-controlled, double-blind trial demonstrated that daily CBL (30 mL/day) is safe, well-tolerated, and feasible for subarachnoid hemorrhage patients, but it does not improve the six-month global functional performance of patients^45^. In the present study, we found that CBL can improve brain injury by alleviating brain edema, neuroinflammation, and neuronal apoptosis after TBI in animals and decrease neuroinflammation in TBI patients.

The mechanisms and molecules regulating neuroinflammation and apoptosis are very complex. The results of the present study showed that CBL can downregulate ROS production, subsequently inhibiting neuroinflammation. Additionally, CBL can also increase the expression levels of TLR2 and TLR4 and alleviate the activation of apoptosis. Barakat *et al.* 46 demonstrated that CBL can improve the neurological score and reduce brain infarction, neuronal degeneration, and leukocyte infiltration by downregulating different elements of the TLR pathway, including TLR-2 and TLR-4, Myd88, TRIF, and IRF-3, and then inhibiting the expression of TLR pathway downstream effectors, including TNF-α, IL-1β, IL-6, and NF-kB. In the intracranial hemorrhage (ICH) model, CBL can ameliorate secondary injury and promote behavioral performance during the acute phase by reducing brain edema, the inflammatory response, and BBB permeability by decreasing the expression levels of IL-1β, TNF-a, IL-6, and AQP4. A recent study also indicated that MCAO rat treatment with a nanoparticle formulation of TLR2shRNA- and TLR4shRNA (T2sh+T4sh)-expressing plasmids can increase the expression of both TLRs 2 and 4, alleviate acute inflammation and improve neurological recovery^47^. This experiment was performed in mice, and debate persists regarding whether the treatment is effective in humans. In the future, we will further explore the clinical effect of CBL on patients with TBI.

## Conclusions

Our study provides evidence that neuroinflammation and apoptosis are mediated by ROS, emerge as an important cellular regulatory mechanism, and contribute to EBI after TBI. In this study, we reported the CBL-mediated regulation of neuroinflammation and apoptosis by the TLR pathway and provided a new idea to explore the biological effects and mechanisms underlying the antineuroinflammatory, antiapoptotic, and neuroprotective properties of CBL.
